# Predictors of short-term mortality after acute stroke in East Azerbaijan province, 2014

**DOI:** 10.15171/jcvtr.2018.06

**Published:** 2018-03-17

**Authors:** Seyed Morteza Shamshirgaran, Hamid Barzkar, Darioush Savadi-Oskouei, Mohammad Yazdchi Marandi, Abdolrasoul Safaiyan, Ehsan Sarbazi, Hossein Novbakht, Saber Gaffari

**Affiliations:** ^1^Epidemiology and Statistics Departement, Faculty of Health Sciences, Tabriz University of Medical Siences, Tabriz, Iran; ^2^Injury Epidemiology Prevention Research Centre, Tabriz University of Medical Siences, Tabriz, Iran; ^3^Neurosciences Research Centre, Imam Reza Hospital, Tabriz University of Medical Sciences, Tabriz, Iran; ^4^School of Nursing of Miandoab, Urmia University of Medical Sciences, Urmia, Iran

**Keywords:** Stroke, Risk Factors, Cause of Death, Mortality

## Abstract

***Introduction:*** Stroke is one of the important causes of death and disability in Iran. This study aimed to examine the factors influencing the short-term mortality of stroke in Northwest of Iran.

***Methods:*** Study population were all patients with confirming the diagnosis of the first-ever stroke who were hospitalized in two referral teaching hospitals from October 2013 to March 2015. They were followed up to 30 days after onset of stroke. A neurology year three resident was responsible for extracting the clinical data and assessment of stroke severity on admission using National Institute of Health Stroke Scale (NIHSS), and information about risk factors and socio-demographic factors were collected using face to face interview. Data were analysed using Cox proportional regression by STATA software version 14.

***Results:*** A total of 1036 consecutive patients with first-ever stroke were included in this study. Of them, 228 patients (22%) died within 30 days after stroke accordance. Advanced age was significantly associated with a hazard for early mortality (HR=1.05 95% CI 1.09–1.04), the inverse was true for education level; mortality decreased as the education level increased; it was 25.7 percent among illiterate and 14.3 among patients with higher education. The NIHSS score on admission for 30-days mortality and hemorrhagic stroke were associated with HR=1.11 (95% CI 1.09–1.13) and HR= 1.65 (95% CI 1.15–2.36) respectively.

***
Conclusion:*** Advanced age, stroke subtype and high NIHSS score are the independent predictors of early mortality in this study. This provides important implications for the clinicians to target the high-risk patients for the specific therapies and management strategies.

## Introduction

Stroke is one of the important cause of death and long-term disability around the world^1^ killing 5.7 million people every year.^2^ Despite the remarkable decline in age-standardized incidence, mortality and disability from stroke, it is predicted that the global burden of stroke has continued to increase. There is a geographical variation in burden of stroke, and the majority of cases are reported from the middle and low-income countries.^3^

In recent years advance progression in management and treatment of acute stroke was achieved. However, it is ranked third leading cause of death in developed countries.^4^ The one-month case fatality rate of stroke varies in different countries; an average of 22.9% has been reported from 13 countries from various parts of the world.^5^

In Iran stroke is one of the most important causes of death and disability. The incidence of stroke has been reported to be from 23 to 103 per 100 000 population^6^ which is lower than developed countries. Previous studies reported 19% to 31% of early mortality of stroke.^7-9^ A recent systematic review and meta-analysis reported 28- days case fatality of 23.6% (95% CI: 17.7-29.5) for a combination of ischemic/hemorrhagic stroke and 13.6% (95% CI: 11.8-15.4%) for ischemic subtype which is higher than most high-income countries.^10^

Recently the conservative treatment approach of stroke has been changed towards to active approach, therefore the focus is now in treatment of the very acute stage of stroke. The identification of early mortality predictors has important implications for clinicians to target the high-risk patients for the specific therapies and management strategies. There is limited information about the early mortality and its predictors in Iran.^8,9^ This study aimed to examine the predictors of short-term mortality after stroke in East Azerbaijan, Northwest of Iran.

## Materials and Methods

This study was conducted in Tabriz, Northwest of Iran and consecutive patients who were admitted to two University referral hospitals; Razi and Imam Reza with a first-ever acute stroke were prospectively included from October 2013 to March 2015. Stroke diagnosis was confirmed when neurological deficits were accompanied by corresponding abnormal findings on brain computed tomography (CT) and/or magnetic resonance imaging (MRI). Patients with symptoms less than 24 hours (TIAs) or those with recurrent stroke were excluded. Neurological and physical examinations, routine blood analyses were performed for all patients. Stroke severity on admission was assessed by the National Institute of Health Stroke Scale (NIHSS).^11^

A neurology resident was responsible for extracting the clinical data including stroke details and other clinical information such as disease history mainly hypertension, diabetes, hypercholesterolemia and heart disease as well as medication history (for checking the presence of comorbidities), positive family history of cerebrovascular diseases. Two trained interviewers were responsible for completing the socio-demographic (age, gender, education, job, income) and lifestyle factors including smoking and alcohol consumption. Alcohol consumption was assessed using one question with “yes/no” answer. Participants were considered themselves as current smoker if they responded “yes” to the question “Do you currently smoke at least one cigarette per day?”

Hypertension (defined as a history of antihypertensive treatment or a history of hypertension – systolic blood pressure [BP] >140 mm Hg, diastolic BP >90 mm Hg, or both); diabetes mellitus (defined by pre-admission history or fasting blood glucose concentration of ≥126 mg/dL after an overnight fast). Information on death was obtained prospectively from the medical records and/or by follow up telephone survey. All deaths occurring within 30 days after stroke were classified as due to stroke; unless an undeniable other cause of death (myocardial infarction, malignancy, car accident, etc) was the obvious cause of death.^12^

Data were analysed by STATA software version 14; quantitative data were expressed as a mean value ± standard deviation (SD), and only the NIHSS score at admission was given as a median value. Univariate analysis using *t* test and chi-square test was performed to show the differences between survivors and deceased group regarding socio-demographic, lifestyle and clinical factors. The Cox proportional-hazards survival regression, which included variables that showed statistical difference (*P* value ≤0.1) on the univariate comparison, was performed.

## Results

A total of 1036 consecutive patients were included in this study. The majority of the study population was male (54.7%), 65 years of age and over (66.2%) and illiterate (67.3%). The mean age was 69.06 ± 12.79. Table 1 shows the general baseline characteristics of the patients with the first-ever stroke according to survival status.

Out of 1036 participants, 228 patients (22%) died within 30 days of their first stroke. Mortality increased as age increased from 7.4% in young age group (<55 years of age) to 28.4% in older patients (75 years and up) (*P* < 0.001). Short-term mortality was about three times higher among hemorrhagic stroke patients compared to ischemic stroke (46.2% vs 16.8% respectively). The inverse was true for education level; mortality decreased as the education level increased; it was 25.7 percent among illiterate patients and only 14.3 among patients with higher education level (*P* < 0.001). Women had a higher mortality than men however it was not statistically significant. The same was true for those who were single/divorced or widow compared to married patients. There was no association between smoking, alcohol consumption and BMI with early mortality (Table 1).

**Table 1:** Socio-demographic characteristic and lifestyle factors among study participants (N = 1036)

**Characteristics**	**Survivors, n=808** **No. (%)**	**Deceased within 30 days n=228** **No. (%)**	***P***
Age group			<0.001
<55	126 (93.6)	10 (7.4)	
55-64	175 (81.8)	39 (18.2)	
65-74	224 (77.2)	66 (22.8)	
75+	283 (71.6)	113 (28.4)	
Sex			
Male	450 (79.5)	116 (20.5)	0.219
Female	358 (76.3)	111 (23.7)	
Education			
Illiterate	518 (74.3)	179 (25.7)	<0.001
Primary school	206 (85.5)	35 (14.5)	
Secondary school & higher‏	84 (85.7)	14 (14.3)	
Marital status			
Married	661 (78.2)	184 ( (21.8)	0.704
Single/divorced/widow	147 (77.0)	44 (23.0)	
Smoking			
Yes	125 (82.2)	27 (17.8)	0.283
No	682 (78.4)	188 (21.6)	
BMI			
<25	362 (75.1)	120 (24.9)	0.001
25-29.9	285 (83.3)	57 (16.7)	
>30	131 (87.3)	19 (12.7)	
Alcohol consumption			
Yes	10 (100)	0 (0.0)	0.132
No	791 (78.6)	215 (21.4)	

Table 2 shows the clinical characteristics of the study participants. Hypertension was the most prevalent clinical condition (85.8%), followed by diabetes (47.8%), heart diseases (27.8%) and positive family history of stroke (20.6%). The median NIHSS score was 6 (range 0–31), and ischemic stroke was the most prevalent subtype of stroke (82.2%). The mean blood glucose on admission was 152.57 ± 76.47 mg/dl. The mean systolic BP at presentation was 144.56 ± 25.80 mm Hg, and the diastolic BP was 84.25 ± 12.86 mm Hg.

**Table 2:** Clinical characteristic of study participants (N=1036)

**Characteristics**	**Survivors (n=808)** **No. (%)**	**Deceased within 30 days (n=228)** **No. (%)**	***P*** ** value**
NIHSS			<0.001
<5	323 (97.6)	8 (2.4)	
6-14	339 (84.1)	64 (15.9)	
15-19	40 (58)	29 (42)	
20+	52 (47.3)	58 (52.7)	
Stroke type			
Ischemic	709 (83.2)	143 (16.8)	<0.001
Hemorrhagic	99 (53.8)	85 (46.2)	
Positive family history			
Yes	184 (86.8)	28 (13.2)	0.001
No	624 (76.2)	194 (23.8)	
Diabetes			
Yes	356 (76.2)	111 (23.8)	0.021
No	420 (82.2)	91 (17.8)	
Heart disease			
Yes	236 (82.2)	51 (17.8)	0.056
No	571 (76.7)	173 (23.3)	
Hypertension			
Yes	682 (77.6)	197 (22.4)	0.104
No	122 (83.6)	24 (16.4)	
Blood glucose on admission, mean (SD), mg/dL	141.97±73.22	143.70±62.7	0.64
Blood pressure on admission, mean (SD), mm/Hg			
Systolic	144.39±26.14	145.19±24.55	0.68
Diastolic	83.79±12.73	85.95±13.23	0.029
NIHSS, mean (SD), score	7.48±6.49	17.00±8.48	<0.001

In univariate analysis, high NIHSS score on admission (*P* < 0.001), and hemorrhagic stroke aetiology (*P* < 0.001), were associated with early mortality. Early mortality rate was increased as the NIHSS score increased from 2.4% in <5 score category to 52.7% in those with NIHSS score 20 and higher. There was no statistical association between other clinical characteristics of study population and early mortality (Table 2).

Cox-proportional hazards survival regression revealed advanced age and a high NIHSS on admission and stroke subtype of hemorrhagic as independent predictors of early mortality. Advanced age was significantly associated with a hazard for early mortality (HR = 1.05 95% CI 1.09–1.04). Also, the NIHSS score on admission was associated with a HR = 1.11 (95% CI 1.09–1.13) for 30-day mortality, and hemorrhagic stroke was associated with a HR= 1.65 (95% CI 1.15–2.36) (Table 3).

**Table 3:** Independent predictors of early mortality in 1036 patients with first-ever stroke

**Variables**	**HR**	**95% CI**	***P*** ** value**
Advanced age	1.05	1.09-1.04	<0.001
High NIHSS score	1.11	1.09-1.13	<0.001
Hemorrhagic stroke	1.65	1.15-2.36	0.006

## Discussion

Stroke is an important cause of death and disability in Iran. In this study, we examined the predictors of early mortality after acute stroke in Northwest of Iran. Our findings revealed that about 22% of study population died within one month after the onset of the first-ever stroke; it was 16.8% for ischemic stroke and 46.2% for hemorrhagic stroke. This is higher than the previous studies in developed countries; Nedelchev et al in 2010 from Switzerland reported 13% one-month case fatality rate for ischemic stroke.^13^ Results from the original Framingham and the Framingham offspring cohorts^4^ showed that 30-days mortality decreased significantly in men (from 23% to 14%), but not significantly in women (from 21% to 20%). A study by Collins et al showed early mortality rate was 7.4 and 18.8% for ischemic and hemorrhagic strokes, respectively.^14^ However, it is similar to the figures in Iraq (22.7%),^15^ Latin America and the Caribbean (19.3 to 26.2%)^16^ and the average of 22.9% reported from 13 countries from various parts of the world.^5^

Previous studies conducted in Iran reported 19% to 31% of early mortality of stroke,^7-9^ it was reported 14.1% for ischemic stroke and 49.1% for hemorrhagic stroke.^8^ Results of a recent systematic review and meta-analysis reported 28- days case fatality of 23.6% (95% CI: 17.7-29.5) for the combination of ischemic and hemorrhagic stroke.^10^

In the current study age, stroke subtype and stroke severity have been the most important influential factors of early mortality. Advanced age was significantly associated with a hazard for short-term mortality (HR=1.05 95% CI 1.09–1.4). The age range of a first stroke in the recent study based on Framingham and the Framingham offspring cohorts was 69–76 years in men and 69–81 years in women.^4^ The same results reported from a study conducted in Switzerland.^13^ Also, Collins et al found that the highest hazard for a 30-day mortality was advanced age (75 years and older).^14^ A nationwide Danish study including patients with ischemic stroke reported that age is an independent predictor of 30 days case fatality.^17^ Studies from Iran also reported the association between age and early mortality of stroke.^8,18^

In the present study, early mortality was associated with stroke type independently; patients with hemorrhagic stroke had an HR=1.65 (95% CI 1.15–2.36) compared to those with ischemic stroke. It is similar to studies from other countries; stroke subtype was also an independent predictor of 30-day case fatality in the study by de Jong et al and Danish study.^19^ Reports from studies conducted in Iran also confirmed the same figures.^7-9^

Our finding showed the NIHSS score on admission was associated with a HR = 1.11 (95% CI 1.09–1.13) for 30-day mortality. Stroke severity on admission is an important and well-established predictor of stroke mortality. Previous studies confirmed the role of stroke severity in stroke mortality.^16^ Stroke severity using the NIHSS score on admission was associated with a HR =1.15 (95% CI 1.05–1.25) for 30-day mortality in a study conducted in Switzerland.^13^

The current study has strengths and limitations. This is the first study of early mortality of stroke and its predictors in East Azarbaijan province in Northwest of Iran which provides valuable information for clinicians. The limitations of being a hospital-based study affect the generalizability of the study. Although these teaching hospitals are the main stroke centres in this province, study population might not be a representative sample of the entire population of this province. Also, patients who die before admission have not been included. Furthermore, our patients might be different from those who admitted to non-governmental institutes or other hospitals regarding socioeconomic status, lifestyle and clinical presentations.

## Conclusion

The present study reports a 22% case-fatality at 30 days after acute stroke in North West of Iran. Advanced age, stroke type, and stroke severity on admission were the independent predictors of early mortality, while laboratory assessment and lifestyle factors were not independently associated with early mortality and might not be considered as prognostic factors in this study. The results of this study provide important implications for clinicians to target the high-risk patients for the specific therapies and management strategies.

## Competing interests

None.

## Ethical approval

The current study was approved by the Ethics Committee of Tabriz University of Medical Sciences (Ethic number TBZMED.REC.1392.227), and at the beginning of the study, informed consent was obtained in written forms from all of the participants.
